# Tissue-Specific Education of Decidual NK Cells

**DOI:** 10.4049/jimmunol.1501229

**Published:** 2015-08-28

**Authors:** Andrew M. Sharkey, Shiqiu Xiong, Philippa R. Kennedy, Lucy Gardner, Lydia E. Farrell, Olympe Chazara, Martin A. Ivarsson, Susan E. Hiby, Francesco Colucci, Ashley Moffett

**Affiliations:** * Department of Pathology, University of Cambridge, Cambridge CB2 1QP, United Kingdom;; † Centre for Trophoblast Research, University of Cambridge, Cambridge CB2 1QP, United Kingdom; and; ‡ Department of Obstetrics and Gynaecology, University of Cambridge, Cambridge CB2 0SW, United Kingdom

## Abstract

During human pregnancy, fetal trophoblast cells invade the decidua and remodel maternal spiral arteries to establish adequate nutrition during gestation. Tissue NK cells in the decidua (dNK) express inhibitory NK receptors (iNKR) that recognize allogeneic HLA-C molecules on trophoblast. Where this results in excessive dNK inhibition, the risk of pre-eclampsia or growth restriction is increased. However, the role of maternal, self–HLA-C in regulating dNK responsiveness is unknown. We investigated how the expression and function of five iNKR in dNK is influenced by maternal HLA-C. In dNK isolated from women who have HLA-C alleles that carry a C2 epitope, there is decreased expression frequency of the cognate receptor, KIR2DL1. In contrast, women with HLA-C alleles bearing a C1 epitope have increased frequency of the corresponding receptor, KIR2DL3. Maternal HLA-C had no significant effect on KIR2DL1 or KIR2DL3 in peripheral blood NK cells (pbNK). This resulted in a very different KIR repertoire for dNK capable of binding C1 or C2 epitopes compared with pbNK. We also show that, although maternal KIR2DL1 binding to C2 epitope educates dNK cells to acquire functional competence, the effects of other iNKR on dNK responsiveness are quite different from those in pbNK. This provides a basis for understanding how dNK responses to allogeneic trophoblast affect the outcome of pregnancy. Our findings suggest that the mechanisms that determine the repertoire of iNKR and the effect of self-MHC on NK education may differ in tissue NK cells compared with pbNK.

## Introduction

Natural killer cells are lymphocytes that function in both immune defense and in reproduction (1, 2). Tissue NK cells differ from circulating NK cells because their phenotype and function are modified by the local microenvironment (3). NK activity is controlled by integration of signals from activating and inhibitory receptors for stress ligands, adhesion molecules, and polymorphic HLA class I molecules (4). These include members of the killer cell Ig-like receptor (KIR), leukocyte Ig-like receptor (LILR), and C-type lectin-like receptor families (5, 6) Peripheral blood NK cells (pbNK) express inhibitory NK cell receptors (iNKR) stochastically, resulting in diverse NK subsets within an individual (7, 8). The uterine mucosa contains abundant uterine NK cells, present in the nonpregnant luteal phase and the first half of pregnancy. They are CD56^bright^CD16^−^NKG2A^+^ with low cytoxicity and are functionally and phenotypically distinct from both CD56^bright^ and CD56^dim^ subsets in pbNK (9–11). A unique feature of decidual NK cells (dNK) is that in pregnancy they are exposed to maternal as well as paternally inherited, allogeneic HLA-C molecules on fetal trophoblast cells (12).

During early placental development, extravillous trophoblast cells (EVT) from the fetus invade into the decidua and remodel maternal spiral arteries to ensure sufficient blood supply to the developing fetus (13). Reduced trophoblast invasion is associated with major complications of pregnancy, including fetal growth restriction and pre-eclampsia (14). EVT express a unique array of MHC class I molecules: HLA-C, HLA-E, and HLA-G, but not HLA-A or HLA-B (15–17). dNK express receptors for these trophoblast HLA class I molecules and are thus potentially capable of mediating allorecognition of the fetus. Additionally, dNK can bind HLA on surrounding maternal cells. For example, most dNK (95%) express inhibitory CD94/NKG2A, which can bind HLA-E on fetal EVT as well as maternal cells (18). LILRB1, expressed by ∼40% of dNK, is an inhibitory receptor for all HLA class I molecules but has a much higher affinity for the dimeric form of HLA-G on EVT (19, 20). Expression of KIR in dNK is also different from expression in pbNK, and the HLA-C–binding KIR show increased expression frequencies in dNK (21).

We have previously demonstrated that some of these maternal NKR–fetal ligand interactions operate in the decidua (12, 15, 19). KIR interactions with HLA-C molecules are of particular interest because KIR can be inhibitory or activating and both KIR and HLA-C are highly polymorphic (22). Each pregnancy is characterized by different combinations of maternal *KIR* and fetal *HLA-C* genes, resulting in variable dNK inhibition or activation. Women homozygous for the *KIR A* haplotype are at increased risk of pregnancy disorders when the fetus has an *HLA-C* allele carrying a C2 epitope (C2), a combination that will functionally result in strongly inhibitory signals to uterine NK cells (12, 23, 24). Conversely, the *KIR B* haplotype, which includes activating KIR2DS1 that binds C2 ligands, is associated with protection from pre-eclampsia and increased birth weight (12, 25). Ligation of KIR2DS1 by C2 stimulates secretion of cytokines, including GM-CSF, which enhances primary trophoblast migration in vitro, suggesting a mechanism for the protective effect of KIR2DS1 (26).

These genetic and functional data show that dNK activation can be regulated by C2 epitopes on EVT to influence placental development. The weaker inhibitory interaction between KIR2DL3 and fetal C1 appears to have no significant effect on pregnancy outcome, emphasizing the importance of KIR2DL1 and fetal C2. However, dNK proliferate in the uterine microenvironment and it is not known how maternal HLA class I expression affects their education and potential to respond. NK cell education depends on interactions between iNKR and MHC class I molecules that fine-tune NK responsiveness so that stronger inhibition during NK maturation leads to more potent NK cells (27–29). In a situation where there is loss of self–MHC class I molecules, as in viral infection, education allows these highly responsive NK cells to detect the altered self. Individuals homozygous for the *KIR A* haplotype possess predominantly inhibitory KIR, including KIR2DL1 that binds strongly to C2 ligands. It follows that these individuals might have an advantage in surviving infection as well as cancer, as they will have more potent NK cells, especially if they also have C2-bearing allotypes.

Do these concepts translate into the situation at the maternal/fetal interface in utero? Evidence in mice suggests that dNK education is imparted by maternal, not paternal, MHC class I molecules (30). This is supported by genetic findings in humans where women who have a *KIR AA* genotype and a fetus with a paternal C2 ligand are at greater risk of pre-eclampsia than when the fetal C2 is derived from the mother (12). The presence of maternal C2 appears protective. In pbNK, the presence of the C1 or C2 epitope affects the function of NK cells expressing the cognate KIR, and it may also affect their frequency of KIR expression, but whether this is true in dNK is unknown (8, 28). NKG2A and LILRB1 can also bind EVT and so may modify the function of dNK through education and during the effector phase. The frequency and distribution of expression of iNKR on dNK are variable and how this modifies functions regulated by KIR/HLA-C interactions is not known. Understanding how maternal and fetal HLA molecules interact with iNKR would enhance our ability to predict high-risk pregnancies. In this study, we have analyzed the expression of five iNKR that bind HLA class I molecules and compared how these regulate the function of specific subsets of dNK and pbNK. Our data reveal fundamental differences in how maternal iNKR are influenced by self–HLA class I molecules to regulate the phenotype, education, and responsiveness of dNK compared with pbNK.

## Materials and Methods

### Primary cells and tissue

Protocols using human tissue were approved by the Cambridge central Research Ethics Committee (study 04/Q0108/23). Informed written consent was obtained from all donors. Total decidual leukocytes (DL) were isolated by enzymatic digestion of maternal decidual tissue from donors undergoing elective termination between 7 and 12 wk of pregnancy, as described previously (10). PBMC were isolated by density centrifugation using Lymphoprep (Axis-Shield) from fresh venous blood obtained at the same gestational age. Genomic DNA for KIR and HLA-C typing was isolated from purified cells using the QIAmp kit (Qiagen).

### Genotyping of KIR and HLA-C

*KIR* and *HLA-C* were typed from genomic DNA by PCR with sequence-specific primers for their presence or absence as described previously (24). *KIR* genes typed were *3DL1*, *3DS1*, *2DL1*, *2DL2*, *2DL3*, *2DL5*, *2DS1*, *2DS2*, *2DS3*, *2DS4*, *2DS5*, and *2DP1*. Typing for *HLA-C* was performed using a similar approach, which allowed all known *HLA-C* group *C1* alleles to be distinguished from *C2* alleles.

### Flow cytometry

Fresh PBMC or decidual cells were washed and resuspended in 100 ml FACS buffer (PBS, 1% FCS) and incubated with 5 mg human γ-globulins (Sigma-Aldrich) for 15 min to block nonspecific binding. Staining with directly conjugated Abs to surface markers was performed for 25 min at 4°C. Abs were CD56 (clone HCD56, BV421, BioLegend), CD3 (UCHT1, allophycocyanin-Cy7, BioLegend) and CD9-PerCP-Cy5.5 (M-L13, PerCP, BD Pharmingen), KIR2DL1 (143211, FITC, R&D Systems), NKG2A (Z199, allophycocyanin, Beckman Coulter), KIR2DL3/L2/S2 (GL183, PE-Cy7, Beckman Coulter), CD122 (Tu27, APC, BD Biosciences), and LILRB1 (HP-F1, PE, Beckman Coulter). For some donors KIR2DL3 alone was stained using clone 180701 (R&D Systems). KIR3DL1 was stained with DX9 conjugated to biotin (BioLegend). Cells were washed with FACS buffer twice followed by staining the near-IR fixable Live/Dead cell dye (Invitrogen) and biotinylated Ab was detected with streptavidin Qdot-605. After washing in FACS buffer, cells were and fixed in 2% paraformaldehyde. For intracellular Ags, cells were treated with FIX & PERM (Life Technologies) and stained for Ki67 (B56, PerCP-cy5.5, BD Biosciences) or c-Myc (9E10, Alexa647, AbD Serotec). Samples were run on an LSRFortessa FACS analyzer (BD Biosciences) and data were analyzed using FlowJo (Tree Star).

### Functional assays

For functional assays, preparations of DL or PBMC were recovered overnight in RPMI 1640 medium, antibiotics, 10% FCS, and 2.5 ng/ml IL-15 (PeproTech). This low dose of IL-15 maintains NK viability without significant activation. DL preparations (>50% dNK) with <5% pbNK contamination (CD56^+^CD9^−^) were used in functional assays. The education status of NK cells was assessed by incubation of 2 × 10^5^ dNK per well with K562 cells for 5 h at an E:T ratio of 5:1. GolgiStop at 6 μg/ml (BD Biosciences) was added for the last 4 h of the incubation. Degranulation was assessed by staining with anti-CD107a in the PerCP-Cy5.5 channel (clone H4A3, BioLegend) when staining dNK subsets as described above.

### Statistical analysis

The Wilcoxon signed rank test was used to compare two groups of paired data, and the Mann–Whitney *U* test was used for comparison of groups of nonpaired data. A nonparametric Friedman ANOVA was used to test for differences between multiple KIR2DL1^+^ subsets with different iNKRs. A *p* value <0.05 was considered statistically significant. Correlation between CD122 and c-Myc expression in dNK subsets was tested by calculating the Spearman rank correlation coefficient.

## Results

### Maternal *HLA-C* genotype affects the frequencies of KIR2DL1^+^ and KIR2DL3^+^ dNK

**FIGURE 1.**
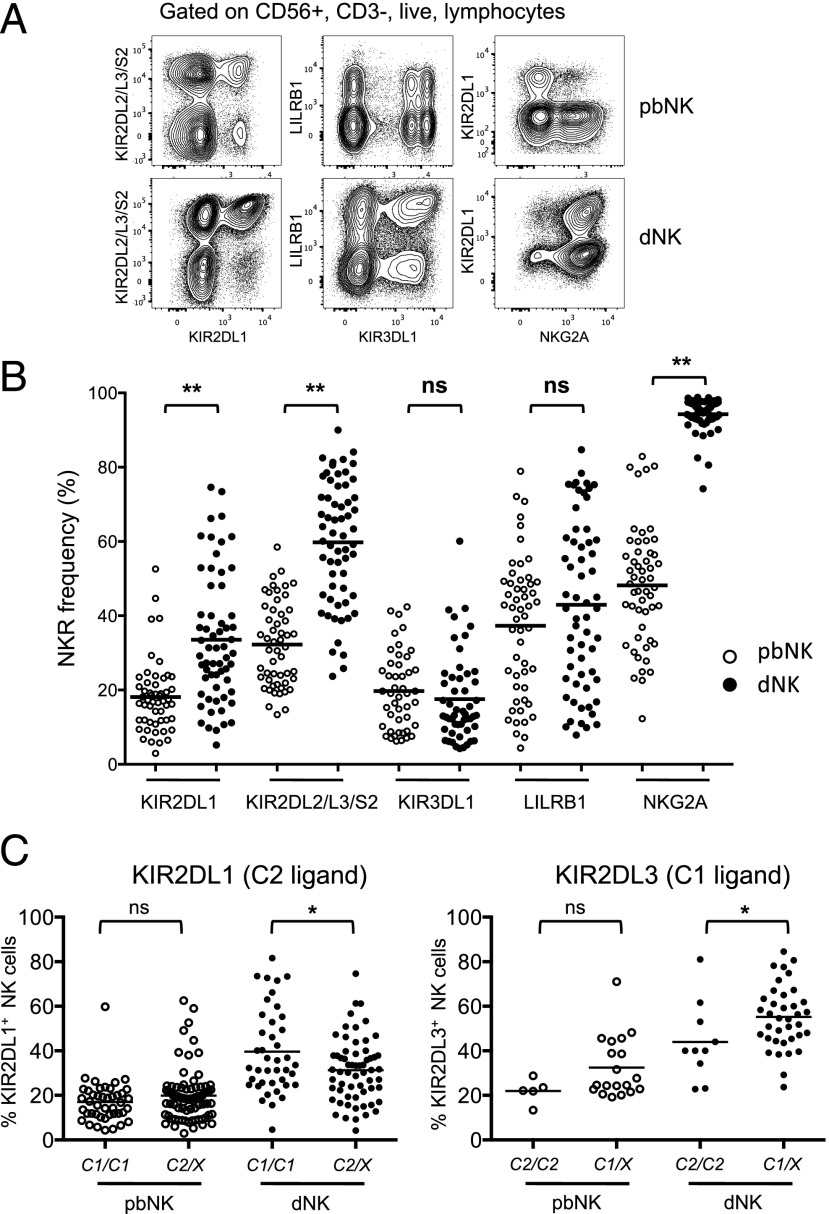
dNK show selective upregulation of specific iNKR compared with pbNK. (**A**) Freshly isolated lymphocytes from decidua or peripheral blood from the same donor were gated on live CD56^+^CD3^−^ cells and examined for expression of KIR2DL1, KIR2DL3, KIR3DL1, LILRB1, and NKG2A. (**B**) Overall frequency of CD56^+^CD3^−^ NK cells positive for the selected iNKRs in samples from blood (○) and decidua (●), expressed as a percentage of total NK cells (*n* = 53 and 61 for pbNK and dNK, respectively). (**C**) Effect of maternal *HLA-C1* or -*C2* alleles on overall frequency of expression of KIR2DL1 and KIR2DL3 was determined by stratifying donors from (B) according to the maternal *HLA-C* type. *C1/C1* indicates donor had two *C1* alleles; *C2/X* indicates either *C2/C1* or *C2/C2* genotype. Frequency of 2DL3^+^ NK cells was determined in pbNK (*n* = 24) and dNK (*n* = 34) by staining with mAb GL183 in patients who were genotyped as KIR2DL2^−^KIR2DS2^−^. *C1/X* indicates donor genotype was either *C1/C1* or *C1/C2*. Horizontal bars indicate the mean percentage, and symbols indicate individual samples. *p < 0.05, **p < 0.001, Mann–Whitney *U* test.

dNK express an unusual repertoire of iNKR compared with their blood counterparts. We used nine-color flow cytometry to examine the expression of selected iNKR (KIR2DL1, KIR2DL3/L2/S2, KIR3DL1, LILRB1, and NKG2A) in samples of freshly isolated dNK and pbNK (Fig. 1A). As previously reported (21), the frequency of KIR2DL1^+^ and KIR2DL3/L2/S2^+^ dNK is increased compared with pbNK, whereas KIR3DL1 is similar (Fig. 1B). NKG2A is expressed on >90% of dNK compared with 48% of pbNK. Expression of LILRB1 is similar for pbNK (mean, 37%; range, 4–79%) and dNK (mean, 43%; range, 8–85%). Analysis of matched pairs of pbNK and dNK from the same donor gave similar results (Supplemental Fig. 1). We examined whether the overall frequency of dNK expressing KIR2DL1 or KIR2DL3 was affected by the mother’s *HLA-C* genotype. The frequency of KIR2DL1^+^ dNK was significantly decreased in mothers with at least one *C2* epitope, the ligand for KIR2DL1 (mean, 31%; range, 4.2–74%) compared with those who were homozygous *C1/C1* (mean, 40%; range, 5–82%; Fig. 1C). In contrast, the frequency of KIR2DL3^+^ dNK was increased in *C1/X* (mean, 55%; range, 24–81%) compared with *C2/C2* mothers (mean, 41%; range, 23–62%; Fig. 1C). Maternal HLA-C status had no significant effect on frequency of KIR2DL1 or KIR2DL3 in pbNK. Thus, unlike in pbNK, the presence of maternal C1 or C2 ligands has different effects on overall frequencies in dNK of their corresponding receptors, KIR2DL1^+^ or KIR2DL3^+^. Because dNK may encounter invading EVT in decidua, we asked whether the presence of fetal *C2* on trophoblast influenced overall KIR2DL1 frequency. Fetal HLA-C genotype had no significant effect (Supplemental Fig. 4).

### Decidual NK cells expressing multiple inhibitory NK receptors proliferate in the decidua

**FIGURE 2.**
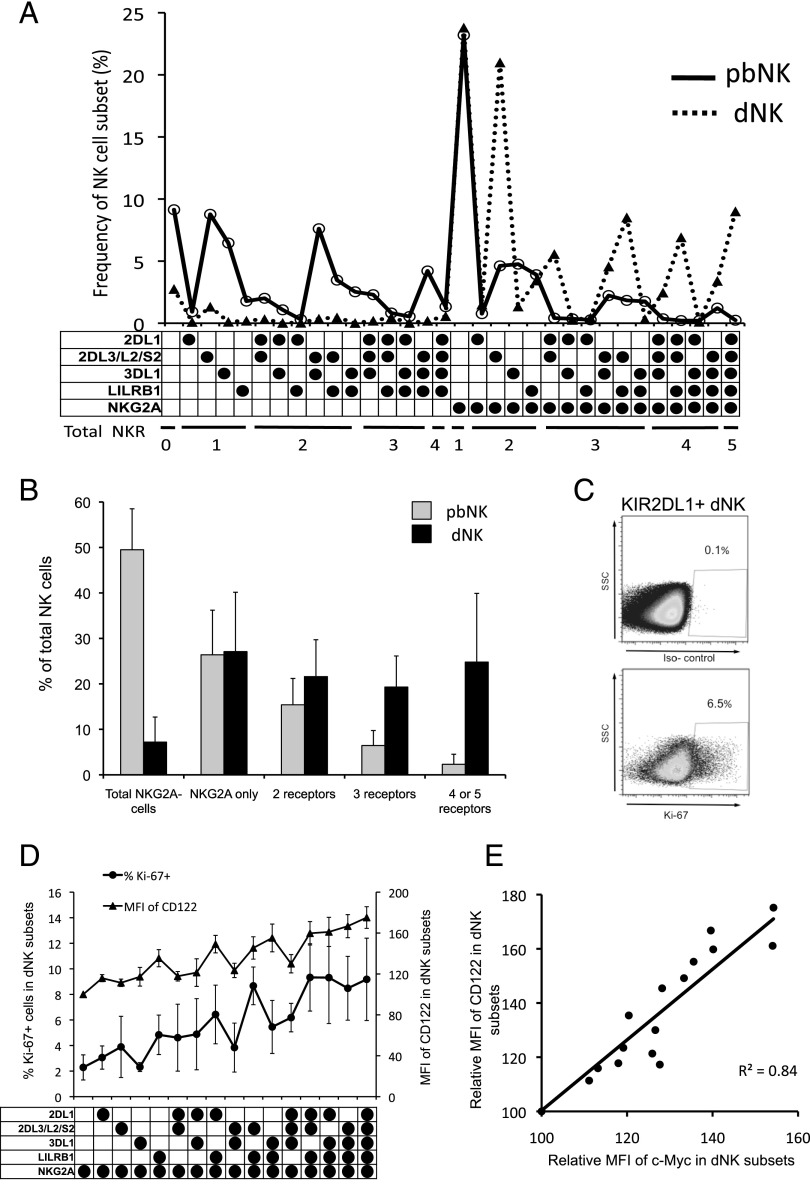
Expansion of specific iNKR subsets in decidual NK cells compared with matched pbNK. The expression of KIR2DL1, KIR2DL3/L2/S2, KIR3DL1, LILRB1, and NKG2A was examined by nine-color flow cytometry in matched pbNK and dNK from 21 donors. (**A**) Thirty-two distinct NK subsets were distinguished on the basis of the five iNKR in paired samples of pbNK and dNK (black and red graphs, respectively). The frequency of each subset from one representative donor is shown as a percentage of total NK cells. This provides a “fingerprint” of the iNKR repertoire, which is characteristic for each donor. The receptor combination for each subset is denoted by black-filled circles, with total number of iNKR in each subset indicated beneath. All NKG2A^+^ subsets are grouped to the right. The donor shown was genotyped as *C1/C1*. (**B**) The proportion of pbNK and dNK that express specific receptor combinations is shown as a percentage of total NK cells (mean ± SD, *n* = 21 matched pbNK and dNK pairs). The proportion of dNK that express two or more iNKR is significantly higher than in pbNK. (**C**) The percentage of dNK cells that are positive for Ki-67 was determined in each iNKR subset by intracellular staining. Control staining used isotype-matched mAb. The NK cells shown were gated on total KIR2DL1^+^ dNK as shown in Fig. 1A. (**D**) The percentages of dNK that are positive by intracellular staining for Ki-67 (●) and for CD122 (▴) are shown for all NKG2A^+^ dNK subsets (mean for each subset indicated by ● or ▴ ± SD, derived from *n* = 11 donors). (**E**) Mean fluorescence intensity (MFI) of staining of each subset is shown following staining for CD122 and c-Myc in the NKG2A^+^ dNK subsets from (D). For each donor, MFI of each subset was expressed relative to the subset expressing NKG2A alone, which was set at 100%. Relative MFI of CD122 and c-Myc is highly correlated (Spearman rank correlation coefficient, *R*^2^ = 0.84, *p* < 0.001).

We next compared the repertoire of KIR2DL1^+^ and KIR2DL3^+^ cells between matched dNK and pbNK samples from 21 donors by analyzing the expression patterns of these five iNKR. This allows quantification of 32 distinct NK subsets, producing a characteristic “fingerprint” for pbNK and dNK from each donor (Fig. 2A). Although the repertoires from each individual are different, the dNK repertoire has distinctive common features. Although most dNK are NKG2A^+^, an equal proportion of pbNK and dNK express NKG2A without other iNKR (∼25%). Notably, 44% of dNK express three or more iNKR compared with only 8.3% of pbNK (Fig. 2B). This dramatic increase is due to expansion of dNK subsets that are KIR2DL3^+^NKG2A^+^ and not simply the higher overall frequency of NKG2A^+^ dNK (Fig. 2A, Supplemental Fig. 2). The frequency of Ki-67^+^ cells in each dNK subset also increases as the number of iNKR rises, suggesting dNK subsets with multiple iNKR expand due to enhanced proliferation (Fig. 2C, 2D). IL-15, a potent mitogen for dNK, is produced by decidualized stroma (31, 32). Intensity of staining for both CD122 (a component of the IL-15 receptor) and c-Myc (a downstream target of IL-15) correlates with Ki-67 staining, implying that dNK subsets with more iNKR expand because of increased sensitivity to IL-15 (Fig. 2D, 2E). Overall, the data suggest clear differences in the effect of maternal HLA-C on the percentage of dNK and pbNK expressing KIR2DL1 or KIR2DL3.

### Decidual NK cells are educated by maternal HLA-C molecules

**FIGURE 3.**
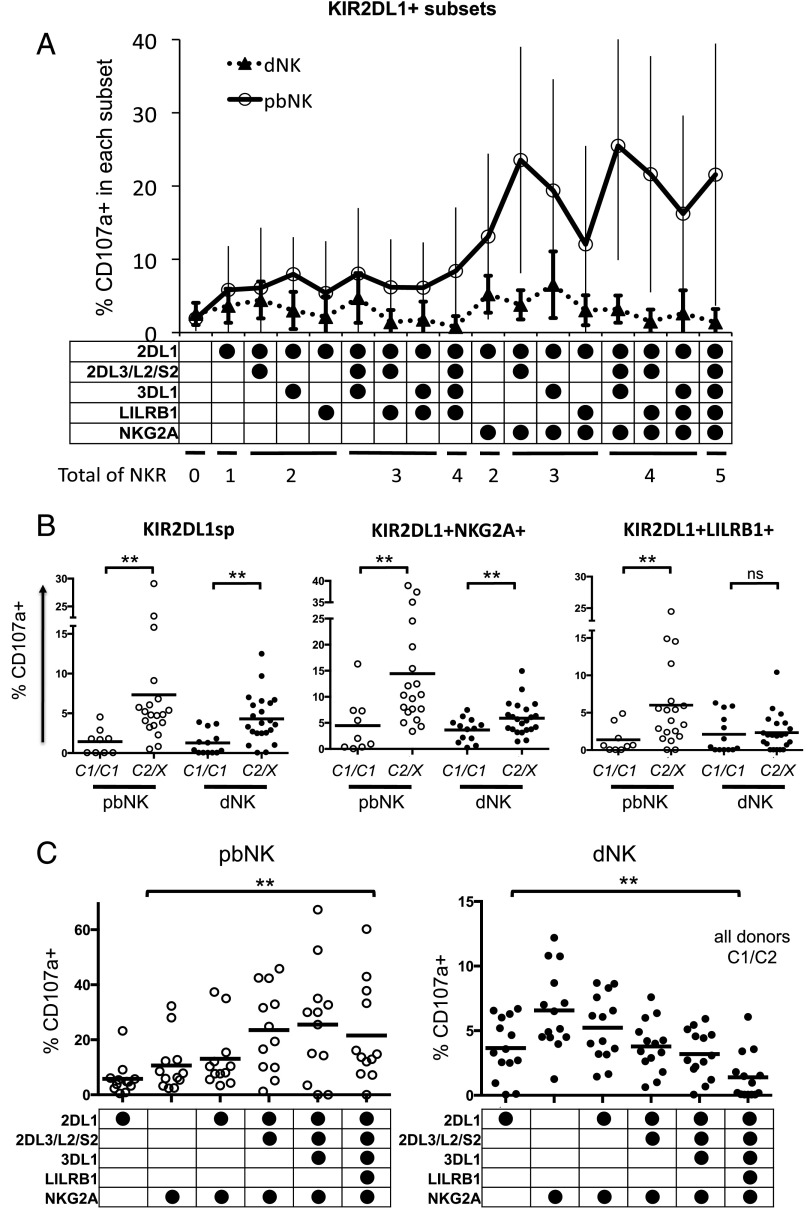
dNK are educated differently by iNKR compared with pbNK. (**A**) Degranulation was measured by CD107a staining of 32 separate NK subsets from pbNK (○) and dNK (●), following coculture with K562 cells (*n* = 28 and 35, respectively). Only KIR2DL1^+^ subsets are shown (see Supplemental Fig. 3 for all 32 subsets). Subsets were defined by expression of five selected iNKRs as described in Fig. 1A. Frequency of CD107a^+^ cells is the percentage of NK cells in that subset that stain positive for CD107a (mean ± SD). Donors were not stratified according to HLA-C genotype. (**B**) Effect of donor’s HLA-C genotype on degranulation in pbNK and dNK in response to K562 was compared between donors stratified according to the maternal HLA-C genotype. *C1/C1* indicates donor homozygous for *C1* group; *C2/X* indicates *C1/C2* or *C2/C2.* Subsets shown are KIR2DL1sp, KIR2DL1^+^NKG2A^+^, and KIR2DL1^+^LILRB1^+^. **p < 0.01, Mann–Whitney *U* test. Bar shows mean. (**C**) Effect of multiple iNKR on degranulation in response to K562 in pbNK (*n* = 12) and dNK (*n* = 14) from C1/C2 donors where both KIR2DL1 and KIR2DL3/L2 will have a maternal HLA-C ligand (●, receptor present on subset). There was a statistically significant difference in degranulation with increasing iNKR on each subset. **p < 0.001 for both pbNK and dNK, Friedman nonparametric ANOVA.

Our genetic findings point to the pivotal role of interactions between maternal KIR2DL1 and fetal C2 epitopes in regulating reproductive success. In contrast, in pregnancies with a C1/C1 fetus, outcomes are independent of KIR genotype. We next asked whether the responsiveness of KIR2DL1^+^ dNK depends on education by maternal HLA-C molecules. We used staining for CD107a to measure degranulation of KIR2DL1^+^ dNK in response to the HLA class I^−^ target cell, K562. This is a robust assay used to measure NK education in human pbNK, which also allows direct comparison of dNK and pbNK (28). In line with previous reports, KIR2DL1^+^ pbNK show increased CD107 degranulation in response to K562 in subsets expressing multiple iNKR (Fig. 3A). This reflects the increased education status of NK cells expressing multiple iNKR for self–HLA class I molecules. In contrast, degranulation of KIR2DL1^+^ dNK was lower than for the corresponding pbNK subset and showed no increase with the number of additional iNKR expressed (Fig. 3A; see Supplemental Fig. 3 for all 32 subsets). To determine whether KIR2DL1 can educate dNK, we compared degranulation of dNK from mothers homozygous for *HLA-C1* alleles (*C1/C1*) to those with at least one *HLA-C2* allele (*C2/X*). KIR2DL1 single-positive dNK (KIR2DL1sp) degranulated more vigorously when the mother was *C2/X*, confirming that maternal C2 epitopes can educate KIR2DL1sp dNK cells as for pbNK (Fig. 3B). Coexpression of KIR2DL1 and NKG2A enhanced degranulation in both dNK and pbNK and preserved the education effect mediated through KIR2DL1 and HLA-C2. In contrast to pbNK, when dNK coexpress KIR2DL1 with either LILRB1 or KIR3DL1, the educating effect of KIR2DL1 was lost (only data for LILRB1 is shown) Fig. 3B). We also studied whether these educating effects are additive by analyzing heterozygous *C1/C2* donors, where education can occur through both KIR2DL1 and KIR2DL3/L2. Degranulation was enhanced in pbNK that coexpressed KIR2DL1 and other iNKR, but not in dNK (Fig. 3C). We conclude that the reduced responsiveness of dNK in subsets expressing multiple NKR occurs because, unlike pbNK, coeducation does not take place.

### The effect of iNKR on NK responsiveness differs between dNK and pbNK

**FIGURE 4.**
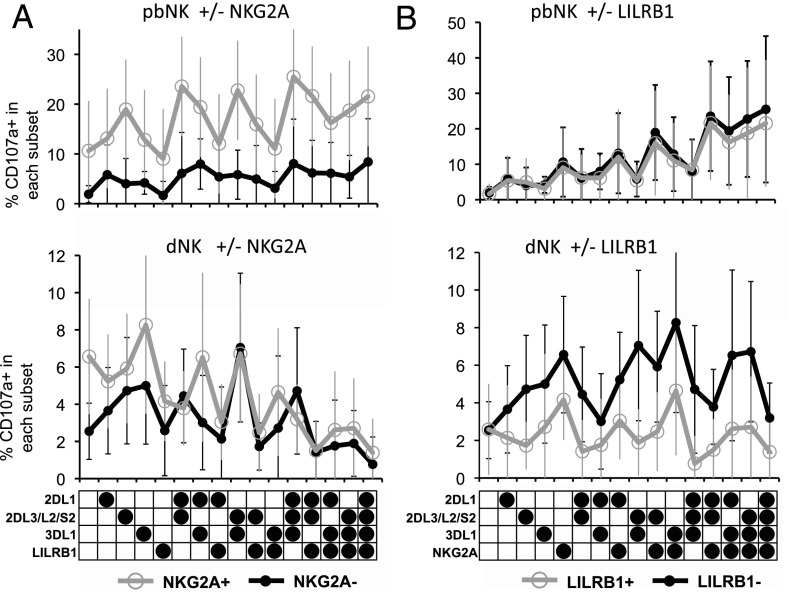
NKG2A and LILRB1 have different effects on responses of pbNK and dNK subsets. (**A**) Effect of presence or absence of NKG2A on degranulation was measured by CD107a staining of 32 separate NK subsets in pbNK and dNK following coculture with K562 cells (*n* = 12 and 14, respectively; all donors genotyped as *C1/C2*). Subsets were defined as described in Fig. 1A. Average frequency of CD107^+^ cells is the percentage of the total NK cells in that subset that stain positive for CD107a (mean ± SD). The response of each subset lacking NKG2A expression (●) is compared with the corresponding subset expressing NKG2A (○). (**B**) The same analysis was performed to compare degranulation of subsets with (○) and without (●) LILRB1 in both pbNK and dNK.

We then determined how NKG2A or LILRB1 modulate responses of all 32 NK subsets in C1/C2 donors. In pbNK, NKG2A has a strong educating effect and all NKG2A^+^ subsets show enhanced responses to K562, compared with the corresponding NKG2A^−^ subset, regardless of how many other iNKR are coexpressed (Fig. 4A). The effect of NKG2A on dNK is more complex. For subsets that express a single additional iNKR, NKG2A enhances responsiveness, but this effect is lost in dNK expressing multiple iNKR. For LILRB1, the opposite effect is observed; coexpression of LILRB1 in subsets with a single iNKR decreases the responses of dNK and the effect persists in subsets with more iNKR. Degranulation of pbNK subsets was unaffected by coexpression of LILRB1 as previously reported (8) (Fig. 4B). In summary, dNK are educated through the interaction of maternal HLA-C with KIR2DL1 and education by NKG2A can enhance these responses. However, unlike pbNK, where LILRB1 has no significant effect, LILRB1 downregulates responses of dNK subsets in which it is expressed and this can overcome any educating effect of KIR2DL1 or NKG2A. Taken together, these results show that dNK differ significantly from pbNK in the way iNKR regulate overall responsiveness.

## Discussion

Pregnancy is unique in that the degree of dNK inhibition depends on both maternal and fetal HLA. Interactions between maternal dNK and fetal HLA-C that result in excessive dNK inhibition are associated with an increased risk of pregnancy complications (12, 23, 24). In this study, we have examined the distribution of five major inhibitory NKR for HLA class I molecules on dNK and analyzed how maternal HLA-C regulates their function. We find there are major differences in how maternal HLA class I molecules regulate dNK responsiveness compared with pbNK, through effects both on the iNKR repertoire and education of dNK.

The primary determinant of iNKR expression in pbNK is genetic. Stochastic promoter regulation leads to an NK cell repertoire with thousands of subsets that can differ even between monozygotic twins (33, 34). KIR gene copy number, host HLA status, and viral infection can impose further changes, including expansion of specific NK subsets (7, 35). The factors determining the repertoire of HLA-binding iNKR in NK cells residing in human tissues (as opposed to blood) remain poorly understood. Our data suggest that selective expansion of certain subsets may contribute to the tissue-specific repertoire seen in dNK. Ki-67 frequency and staining intensity for CD122, the β-chain of the IL-15 receptor, were highest on subsets with multiple iNKR, which also showed upregulation of c-Myc. Higher CD122 may increase sensitivity to IL-15, a cytokine that stimulates vigorous dNK cell division and acquisition of KIR (36, 37). Selective expansion of dNK subsets that coexpress KIR2DL3 and NKG2A was also found, but because these subsets did not show increased CD122, other mechanisms must regulate their expansion.

When donors were stratified based on the presence of C1 or C2 epitopes, we found that KIR2DL1 frequency was significantly decreased in dNK in C2 mothers whereas KIR2DL3 increased in dNK in mothers with C1. This contrasts with pbNK, where some studies have reported increased expression of KIR2DL1 in pbNK of C2 individuals, whereas others have found no effect (38–40). We found no evidence that fetal *HLA-C* genotype affected KIR in dNK. Why KIR2DL1 and KIR2DL3 frequencies in dNK should be differently affected by their maternal HLA ligands is not clear. The reduction in frequency of KIR2DL1^+^ dNK by maternal C2 epitopes could be an evolutionary adaptation to reduce risk in pregnancies where the chance of an HLA-C2 fetus is at least 50% and help to maintain *HLA-C* alleles with C2 epitopes in the human population (22).

Because the overall inhibition experienced by dNK appears to regulate placentation, a key aim of this study was to determine how the main iNKRs for HLA class I molecules regulate dNK responsiveness. KIR2DL1 has previously been shown to educate pbNK through C2, and we show in the present study that KIR2DL1 also educates dNK upon interaction with maternal C2 (28, 41). Owing to the scarcity of relevant donors, we were unable to determine whether dNK are also educated by KIR2DL3 binding to maternal C1 epitopes. Collection of sufficient *KIRAA* mothers with *C2/X* or *C1/C1* genotypes is ongoing. dNK proliferate vigorously in the decidua while expressing a full complement of KIR and NKG2A. Because these freshly produced dNK are educated by HLA-C, this suggests that adaptation to the maternal HLA environment takes place locally in the decidual tissue. These findings are similar to those in the mouse where maternal but not paternal MHC ligands educate NK cells in the decidua (30).

An important finding is that there are major differences in how iNKR combine to regulate responsiveness of dNK compared with pbNK. In pbNK, NKG2A is a strong educating iNKR, and all NKG2A^+^ subsets degranulate more vigorously in response to K562 than does the corresponding subset without NKG2A (8). In dNK the effect of NKG2A was more complex. NKG2A maintained the educational effects of KIR2DL1 to maternal C2, but in subsets expressing additional iNKR, responses were decreased. In contrast, LILRB1 had little effect on pbNK responsiveness, but in all dNK subsets it abolished the educational effects of KIR2DL1 and decreased responsiveness. This may be due to the greatly increased avidity of binding of LILRB1 to the dimeric form of HLA-G found on EVT, because other LILRB1/HLA interactions remain similar in pbNK and dNK (19). In pbNK we find that as the number of educating iNKRs on the subset increases, responsiveness goes up due to additive effects of education, in agreement with previous reports (8, 42). In dNK the reverse is true; responsiveness decreases in subsets with more “educating” iNKR. The overall effect of interactions between KIR, NKG2A, and LILRB1 is that dNK degranulation in response to K562 remains similar regardless of the number of iNKR. We conclude there are fundamental differences in the ways that iNKR educate individual subsets in pbNK and dNK. A limitation of this study is that dNK responsiveness was assessed by degranulation alone. Whether secretion of cytokines by dNK is similarly affected by NKR remains to be determined, but in pbNK the effects of NKR on degranulation and IFN-γ secretion were similar (8, 28).

Few studies have examined the repertoire and functions of inhibitory MHC receptors in NK cells from nonlymphoid human tissues. Although heterogeneous, NK cells in other human tissues share some characteristics of dNK. They tend to be CD56^bright^, NKG2A^+^, produce a variety of cytokines, and are less cytotoxic than pbNK (3). In the liver ∼50% of the NK cells are NKG2A^+^, and they are educated by KIR that bind self-HLA (43). In fetal tissues, up to 90% of NK cells are NKG2A^+^. Fetal NK cells are educated by NKG2A but not by KIR binding to self-HLA; instead, KIR^+^ fetal NK cells are hyporesponsive (44). Our finding that some dNK are more responsive when they are NKG2A^+^ suggests that this NKR may also educate NK cells in the decidua, but the educating effect was not seen in all subsets (Fig. 4A). An alternative explanation is that NKG2A does not educate dNK but is a marker for more activated NK cells in decidua. However, all dNK are CD69^+^,so there is currently no evidence for differential activation among dNK subsets (10). These studies emphasize that the functional consequences of iNKR interactions with the cognate HLA ligands in primary NK cells are likely to be tissue specific. Predicting effects of specific iNKR–HLA interactions on repertoire and NK function based on results from pbNK may be misleading. This is particularly relevant as clinical trials begin with mAbs targeting iNKR.

Our genetic findings in several cohorts indicate that the key effects of HLA-C in pregnancy are mediated by KIR that recognize C2 epitopes (12, 23–25). The interaction between KIR2DL3 and fetal C1 appears to have no significant effect on pregnancy outcome. The contrasting effect of maternal HLA-C on KIR2DL1 and KIR2DL3 expression in this study highlights their different roles in placentation. In the present study, we have shown that dNK functionally adapt to the presence of maternal C2 though education in the decidua as well as by reducing the frequency of cells expressing KIR2DL1. Both findings suggest mechanisms whereby maternal HLA-C2 can modify the risk of abnormal placentation in *KIR AA* mothers with an HLA-C2 fetus (12, 24). However, how education by maternal C2 actually alters dNK function in a manner beneficial to pregnancy remains to be elucidated. Finally, we have also shown that there are significant differences in how KIR2DL1 and other iNKR combine to educate NK cells in decidua compared with pbNK. Whether these differences in education of NK cells occur in other tissues will clearly be important in understanding NK function in diseases such as cancer.

## Supplementary Material

Data Supplement
