# Supplementary material for: Tissue-Specific Education of Decidual NK Cells
Source: J Immunol. 2015 Aug 28;195(7):3026–32. doi: 10.4049/jimmunol.1501229 (PMC4574523; doi:10.4049/jimmunol.1501229)
Supplement: Data Supplement [file JI_1501229.zip › JI_1501229_Supplemental_Figures_1.pdf]

Supplementary Figure S1

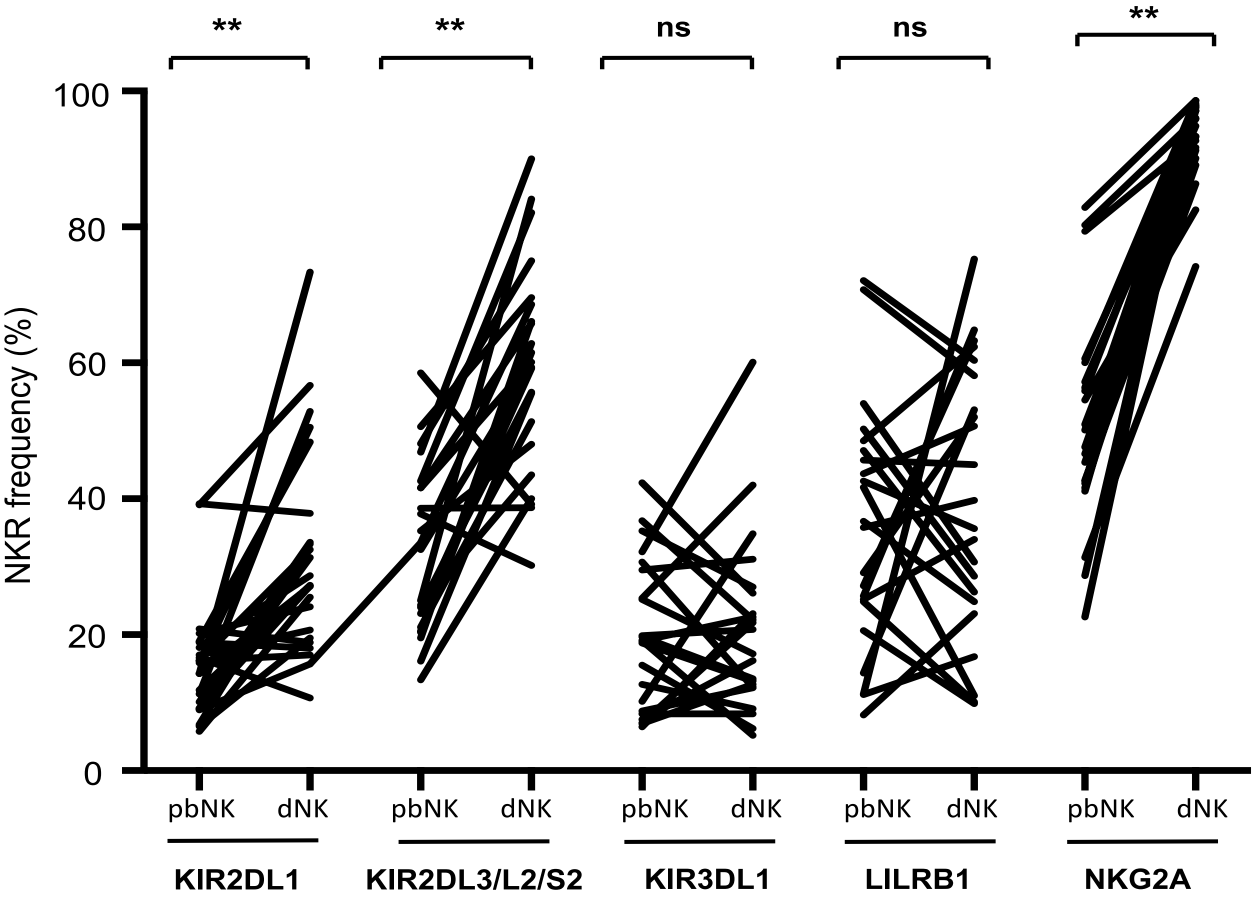

Supplementary Figure S1.

dNK show selective upregulation of specific iNKRs compared to pbNK from the same donor.

(A) Overall frequency of $CD56^{+}CD3^{-}$ NK cells positive for the selected iNKRs in paired samples from blood and decidua, expressed as a percentage of total NK cells (n=21). Cells were gated as shown in Figure 1A. ** indicates p<0.01, Wilcoxon signed rank test; ns, not significant.

Supplementary Figure S2

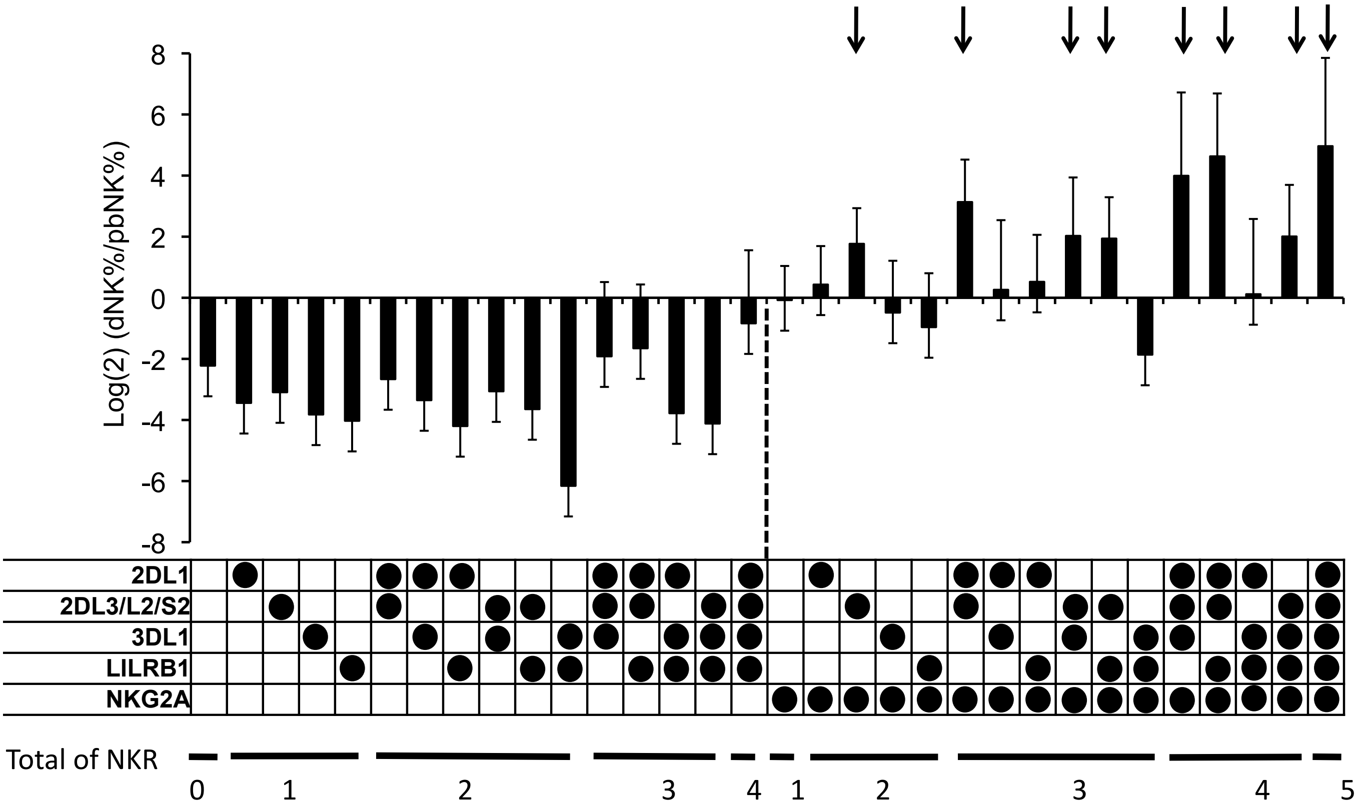

Supplementary Figure S2

Subsets that co-express KIR2DL3 and NKG2A are selectively expanded in dNK compared to dNK.

For each donor the frequency of each subset in dNK and pbNK was expressed as a ratio (dNK/pbNK, n=21, matched pairs). The log2 mean ratio (± SD) for each subset is shown. This indicates whether on average, the subset was increased or decreased in dNK compared with pbNK from the same donor. The receptor combination for each subset is denoted by black filled circles. Subsets that co-express KIR2DL3 and NKG2A are indicated with arrows and are increased in frequency in dNK compared to pbNK from the same donor.

Supplementary Figure S3

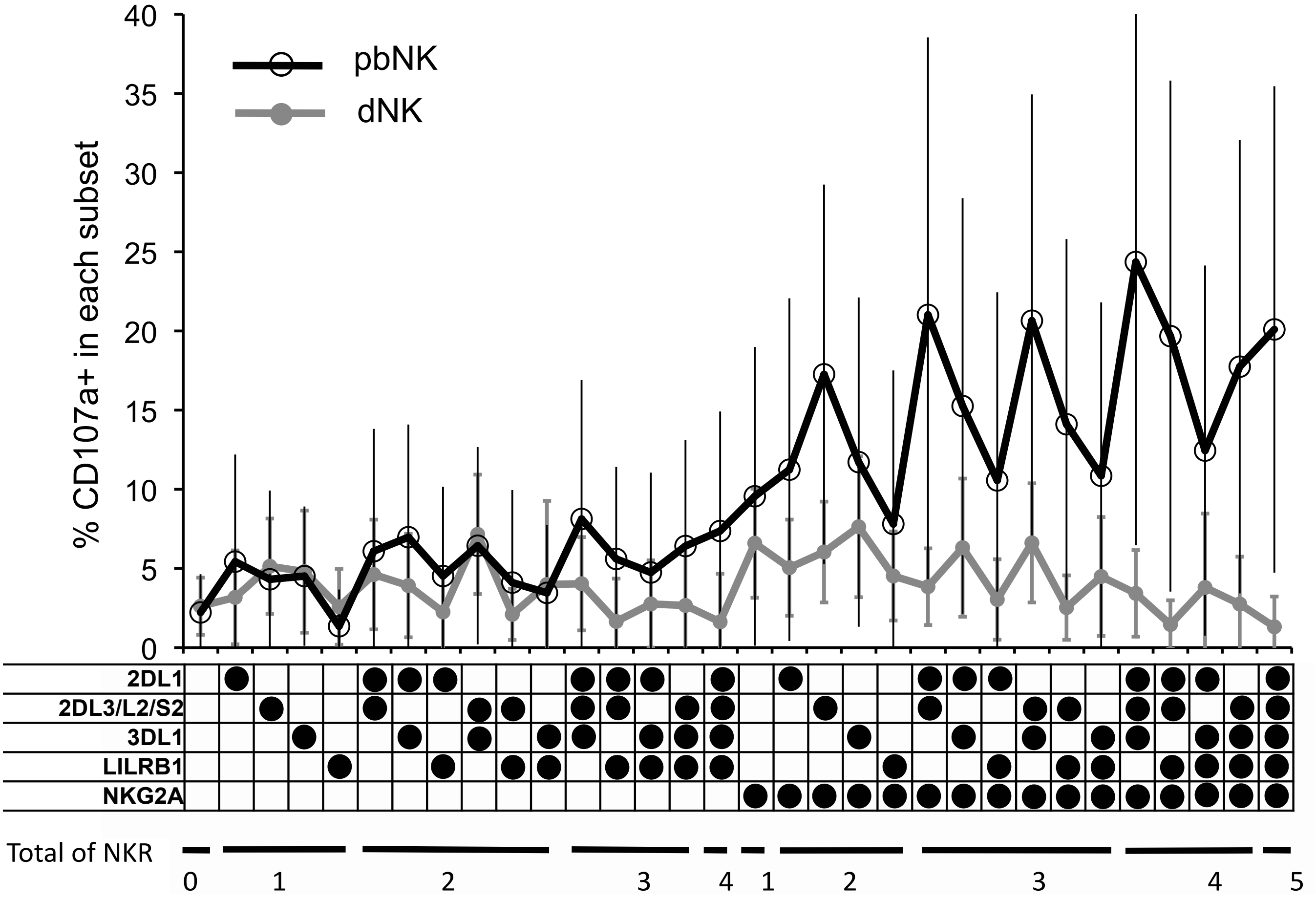

Supplementary Figure S3

Effect of iNKRs on responsiveness of pbNK and dNK cells to K562 cells is very different.

Degranulation was measured by CD107a staining of 32 separate NK subsets in pbNK and dNK following co-culture with K562 cells (n=28 and 35 respectively). Subsets were defined by expression of 5 selected iNKRs as described in Figure 1A. Mean frequency of CD107a+ cells is the percentage of the total NK cells in that subset that stain positive for CD107a (mean +/- SD). The receptor combination for each subset is denoted by black filled circles. Co-expression of iNKRs has different effects on responsiveness of each subset in matched pbNK and dNK

Supplementary Figure S4

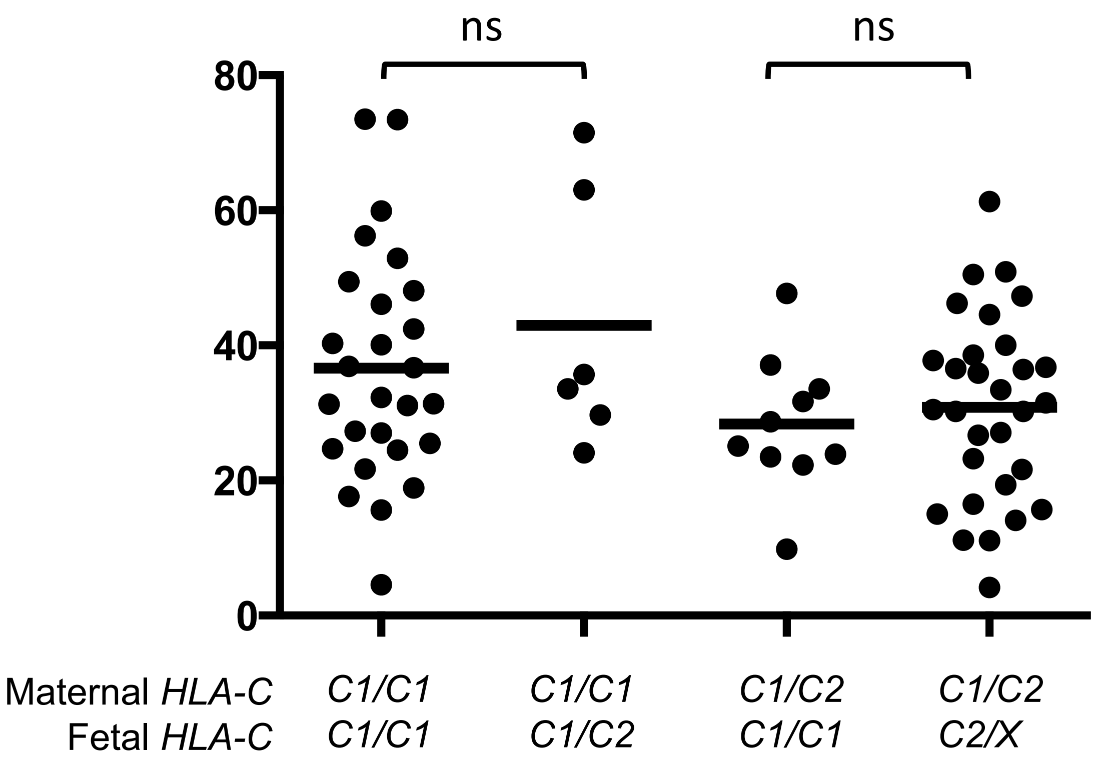

Supplementary Figure 4.

Fetal *HLA-C type does not significantly affect overall frequency of expression of KIR2DL1 in maternal dNK.*

Overall frequency of KIR2DL1+ dNK cells was determined by FACS as described in Figure 1 and stratified according to the maternal and fetal HLA-C type. Horizontal bar indicates mean percentage. *C1/C1* indicates donor had both alleles of *C1* group, *C2/X* indicates either *C2/C1* or *C2/C2*. Presence of fetal *C2* had no significant effect on KIR2DL1+ dNK frequencies in *C1/C1* or *C1/C2* mothers, Wilcoxon signed rank test; ns, not significant.
